# Extracellular Volume Fraction Analysis on Cardiac Computed Tomography Is Useful for Predicting the Prognosis of Hypertrophic Cardiomyopathy

**DOI:** 10.3390/jcdd12090372

**Published:** 2025-09-19

**Authors:** Shuhei Aoki, Hiroyuki Takaoka, Tomonori Kanaeda, Kazunari Asada, Joji Ota, Yoshitada Noguchi, Moe Matsumoto, Yusei Nishikawa, Katsuya Suzuki, Satomi Yashima, Makiko Kinoshita, Noriko Suzuki-Eguchi, Haruka Sasaki, Kohei Takahashi, Yoshihito Ozawa, Yosuke Inaba, Yoshio Kobayashi

**Affiliations:** 1 Department of Cardiovascular Medicine, Graduate School of Medicine, Chiba University, Chiba 260-8677, Japan; cdwa0004@chiba-u.jp (S.A.);; 2 Department of Cardiovascular Medicine, Eastern Chiba Medical Center, Togane 283-8686, Japan; 3 Department of Radiology, Chiba University Hospital, Chiba 260-8677, Japan; 4 Biostatistics Section, Clinical Research Center, Chiba University Hospital, Chiba 260-8677, Japan

**Keywords:** hypertrophic cardiomyopathy, extracellular volume, computed tomography

## Abstract

Extracellular volume fraction (ECV) analysis on computed tomography (CT) is now available. The purpose of this study was to assess the usefulness of CT-derived ECV analysis for predicting outcomes in patients with hypertrophic cardiomyopathy (HCM). One hundred and one HCM patients (67 males, 66 ± 11 years old) who received cardiac CT between January 2009 and December 2021 were included. We measured left ventricular (LV) ECV (LV-ECV) on CT and investigated the relationship between LV-ECV and the major adverse cardiac events (MACE) after CT. Fifteen patients (15%) experienced MACE. The patients with MACE had a significantly higher LV-ECV, left atrial diameter, LV end-systolic diameter, and lower LVEF than those without MACE. The proportion of dilated phase HCM was significantly higher in the patients with MACE than those without MACE. LV-ECV and LVEF were significant predictors of MACE based on the multivariate analysis by Cox proportional hazards model. The optimal threshold of LV-ECV to predict MACE was 37.6% based on the receiver operating characteristic analysis. The patients with LV-ECV ≥ 37.6% (30 patients) experienced significantly higher MACE than those with LV-ECV < 37.6% (*p* < 0.001). CT-derived ECV analysis suggested potential usefulness for predicting MACE in patients with HCM.

## 1. Introduction

Hypertrophic cardiomyopathy (HCM) is recognized as a significant cardiovascular disorder that may result in life-threatening ventricular arrhythmia, syncope or sudden cardiac death, including among younger individuals [1]. The usefulness of transthoracic echocardiography (TTE) and magnetic resonance imaging (MRI) for risk stratification and the detection of high-risk patients with HCM is already known broadly [2,3].

Detection of late gadolinium enhancement (LGE) using cardiac MRI has been the gold standard in the qualitative evaluation of myocardial fibrosis in patients with cardiomyopathies. The recognition of the LGE pattern is useful for the differential diagnosis of several types of cardiomyopathies, and the presence of LGE is a marker of worse prognosis in several myocardial diseases, including HCM [3]. Additionally, T1 mapping analysis on MRI is now available, and evaluation of native T1 values and extracellular volume fraction (ECV) using this mapping is also helpful in the quantitative analysis of myocardial fibrosis [4].

However, MRI has some limitations. MRI can be limited in individuals with non-MRI-conditional cardiac mechanical devices due to safety concerns. Furthermore, MRI is not suitable for individuals with severe claustrophobia, and patients with hemodialysis are not candidates for gadolinium contrast for LGE analysis. Even if cardiac mechanical devices are acceptable for MRI, obtaining clear images is not easy. This is due to frequent device checks before and after image acquisition and lower image quality caused by mechanical artifacts. Moreover, LGE imaging is complex in patients with arrhythmia and heart failure because of motion artifacts or difficulty holding their breath [5]. In contrast, cardiac computed tomography (CT) has fewer restrictions compared to MRI and can be used for more patients. Particularly in HCM patients, who often have ST abnormalities on ECG or chest discomfort, cardiac CT is commonly used to rule out coronary artery disease. Based on recent guidelines, cardiac CT is recognized as a valuable tool for screening coronary artery stenosis in individuals suspected of having myocardial dysfunction [6]. In addition, recent advancements in scanners enabled the acquisition of clear images even in patients with arrhythmia [7]. If late-phase scans are performed, they are helpful for screening intracardiac thrombus and detecting myocardial fibrosis occurring as late iodine enhancement (LIE), similar to the LGE [8]. Additionally, the ECV of left ventricular myocardium (LVM) (LV-ECV) is evaluable on those images, and the ECV values measured by CT correlate well with those of MRI [9].

Following recent reports on the utility of ECV analysis on MRI in predicting ventricular arrhythmia in cases with HCM [10], we speculated on the presence of a correlation between LV-ECV on CT and adverse events in HCM patients. To investigate this potential association, we evaluated the utility of CT-derived ECV analysis for predicting adverse events in the study cohort.

## 2. Materials and Methods

This study was a multicenter retrospective cohort study. This retrospective study enrolled HCM patients who underwent cardiac CT at Chiba University Hospital and Eastern Chiba Medical Center between January 2009 and December 2021. Patients were included if they underwent cardiac CT with late-phase acquisition using tube voltage consistent with non-contrast scanning protocols and completed a follow-up period of more than one month. Two cases were excluded because ECV analysis could not be performed due to significant gaps in the LVM images between the non-contrast and late-phase cardiac images. Patients who underwent CT at Chiba University Hospital between January 2016 and June 2020 were excluded because ECV analysis could not be performed due to different tube voltages between the non-contrast and late-phase cardiac images. We measured CT-derived ECV on LVM and collected comprehensive data, including patient characteristics, transthoracic echocardiographic findings, and other CT parameters. The relationship between these collected data and the occurrence of major adverse cardiac events (MACE) following the CT scan was subsequently investigated. MACE were defined as a composite of cardiac death, fatal arrhythmia, and heart failure hospitalization. The observation period was set until March 2023.

We compared patient background characteristics between the groups with and without MACE and assessed previously identified important factors for predicting MACE in HCM patients [11]. HCM was defined by cardiac findings demonstrating unexplained left ventricular hypertrophy (LVH) measuring ≥ 15 mm (or ≥13 mm for individuals with a family history of HCM) [12,13]. Patients with secondary causes of left ventricular hypertrophy, specifically hypertension, Fabry disease, amyloidosis, mitochondrial disease, or congenital heart disease, were excluded. Patient background data, such as risk factors for coronary artery disease and administered medical treatments, were acquired from Chiba University Hospital and Eastern Chiba Medical Center. The risks of sudden cardiac death of patients were evaluated based on the previous study [14]. The institutional review board of Chiba University approved this retrospective analysis (reference number is 3822).

### 2.1. Protocol for Computed Tomography

CT examinations were conducted using either a 256-row detector CT scanner (Revolution CT Apex, GE Healthcare, Waukesha, WI, USA) or a 320-row detector CT scanner (Aquilion One or Aquilion One ViSion Edition, Canon Medical Systems, Otawara, Japan). Tube current for all scans was determined by the automatic exposure control system. A non-contrast ECG-gated cardiac scan, performed with a prospective ECG-gated technique, was acquired before the contrast administration. For the non-contrast scan, slice thickness was 0.5 mm and tube voltage was set to 100 or 120 kV on the 320-row CT, while the 256-row CT used a slice thickness of 0.625 mm and a tube voltage of 120 kV.

The early phase scan for coronary artery evaluation was performed using retrospective ECG gating, with dose modulation applied to reduce radiation exposure. As previously reported, conventional enhanced CT was conducted using a slice thickness of 0.5 mm and a tube voltage of 120 kV [15]. If a patient’s heart rate was ≥65 beats per minute, 20 mg of metoprolol tartrate or 12.5 mg landiolol was administered before the scan, assuming no contraindications to β-blocker therapy existed. Two doses of sublingual isosorbide dinitrate were given immediately before scanning.

Contrast material was administered using a triphasic protocol, which was previously identified as providing higher diagnostic accuracy for detecting coronary artery stenosis on CT [16]. Intravenous access was secured in the right or left antecubital vein with a 20 or 22-gauge needle. A dual-syringe injector featuring a dual-flow option (Dual Shoot GX7, Nemoto, Tokyo, Japan) was utilized for administration. The protocol involved a first phase injecting 50–110 mL of undiluted iodinated contrast agent (300–370 mg/mL), followed by 0–50 mL of a 50% saline-to-contrast mixture. 

A late-phase scan was performed 6 min after the injection of iodinated contrast medium, utilizing the prospective ECG-gating technique [17]. The CT scan was conducted using the same slice thickness and the same tube voltage as in the non-contrast scan.

This protocol is consistent with that used in our previous study [15].

### 2.2. Analysis of Cardiac CT Images Including ECV

**Figure 1:**
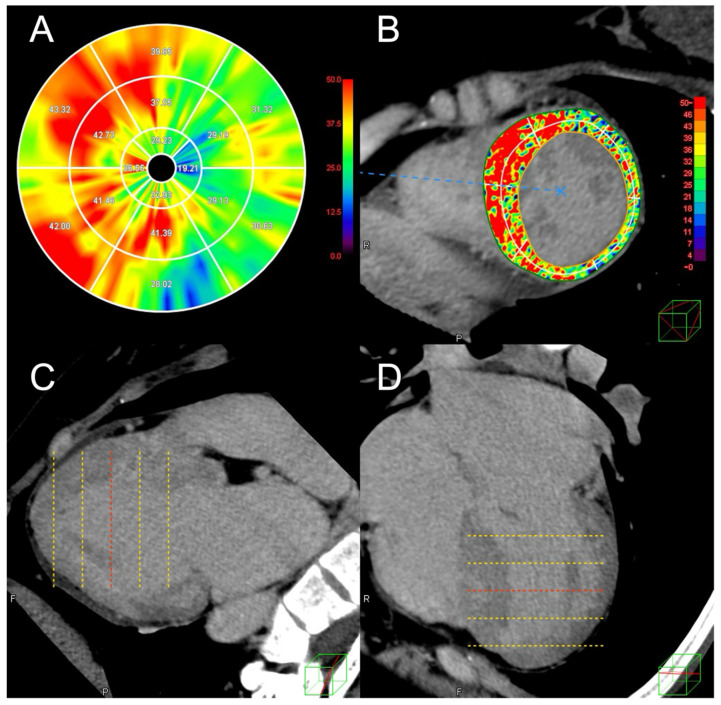
Extracellular volume analysis of left ventricular myocardium on computed tomography images in a case with hypertrophic cardiomyopathy. (**A**) A polar map displaying the segmental extracellular volume fraction (ECV) of left ventricular myocardium for each of the 16 American Heart Association segments. (**B**) A short-axis view with a color-coded ECV map overlaid. (**C**) Two-chamber and (**D**) four-chamber views of the late-phase computed tomography (CT) images used for the analysis. This case is of a 28-year-old female with HCM who was hospitalized for heart failure after the CT scan. The overall ECV of the left ventricular myocardium on CT was 37.6%.

ECV of the LV myocardium was calculated using the formula ECV = (ΔHUm/ΔHUb)/(1 − Hct) with commercially available software (Ziostation 2, Ziosoft Inc, Japan). In this formula, ΔHUm indicates the change in myocardial CT attenuation (in Hounsfield units), ΔHUb the change in blood CT attenuation, and Hct the hematocrit [17]. An automatic three-dimensional non-rigid registration of the myocardium was performed between the non-contrast and late-phase CT images to generate subtraction images, facilitating ECV calculation. From these images, the change in CT attenuation (ΔHU) was measured [17,18]. A polar map illustrating the mean ECV value for each of the 16 American Heart Association LVM segments was generated (Figure 1A). LV-ECV measurements on CT were conducted by a board-certified cardiologist (Hi.T.) with 15 years of cardiac CT reading experience and another cardiologist (S.A.) with four years of experience. These observers also assessed for the presence of LIE of LVM on the same workstation, resolving any disagreements through a consensus reading. Sample images demonstrating the ECV analysis on CT are shown in Figure 1.

**Figure 2:**
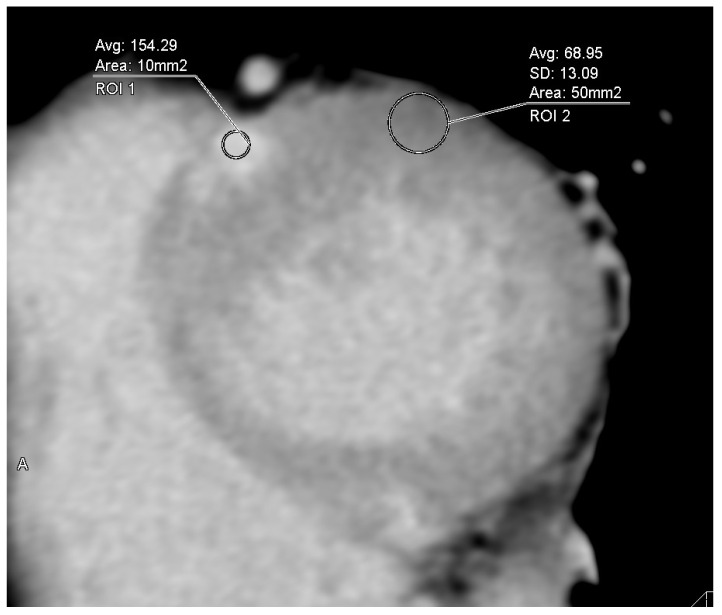
Evaluation of contrast-to-noise ratio in late-phase cardiac computed tomography images. Quantitative image quality analysis was performed by manually drawing approximately 0.1 cm^2^ regions of interest on the late iodine enhancement (LIE) and 0.5 cm^2^ on the remote myocardium in the cases with LIE of left ventricular myocardium on computed tomography (CT). Contrast-to-noise ratio (CNR), an indicator of image quality, was also quantified. CNR is obtained by dividing the difference in CT attenuation between late enhancement area and normal myocardium by the standard deviation of the normal myocardium CT attenuation.

For quantitative image quality analysis, regions of interest were manually drawn, approximately 0.1 cm^2^ in areas of late iodine enhancement (LIE) and 0.5 cm^2^ in the remote myocardium, in cases where LIE of LVM was present on CT (Figure 2). The contrast-to-noise ratio (CNR), an indicator of image quality, was also quantified. CNR is obtained by dividing the difference in CT attenuation between the late enhancement area and the normal myocardium by the standard deviation (SD) of CT attenuation in normal myocardium [15,18]. CNR was evaluated in all 40 patients (80%) with LIE on CT (by S.A.).

Significant coronary artery stenosis of >70% (>50% in the left main coronary artery) in 1 or more major epicardial coronary arteries, was evaluated based on the 15 segments model of the American Heart Association [19,20]. Coronary artery segments measuring at least 1.5 mm in diameter were all evaluated. The significance and position of the stenosis was assessed by them using the same workstation. In cases of disagreement between the two observers, a consensus reading was performed to resolve the issue.

### 2.3. Echocardiographic Measurement

TTE was conducted as part of routine clinical practice, employing EPIQ 7G and X5-1 transducers (Philips Medical Systems, Andover, MA, USA), iE33 and S5-1 transducers (Philips Medical Systems, Andover, MA, USA), Vivid E-9 and M5-Sc-D (GE Healthcare, Milwaukee, WI, USA) or Aplio 400 and PST-25BT transducers (Canon, Tokyo, Japan) by standard methods according to the recommendations of the European Society of Cardiology [21,22]. In parasternal long-axis views, LV diameters were assessed using direct two-dimensional (2D) measurements at both end-diastole and end-systole. LV ejection fraction (LVEF) was assessed using the Teichholz or biplane disk summation method. Additional standard measurements of cardiac size, function, and LV wall thickness were performed in accordance with the European Society of Cardiology [21].

The LV outflow tract (OT) flow was visualized in the apical long-axis color flow image, and a continuous wave Doppler cursor was directed toward the LVOT flow. The Doppler beam was assumed to be almost parallel to systolic flow in the LVOT. After that, the peak flow velocity and LVOT pressure gradient was estimated by utilizing the modified Bernoulli equation.

Significant valvular abnormality was defined as the presence of ≥moderate valvular stenosis or regurgitation [15].

### 2.4. Statistical Analysis

Continuous variables were expressed as mean ± standard deviation, and categorical variables in numbers and percentages. Welch’s t-test was used for continuous variables, and Fisher’s exact test was used for categorical variables. Patient backgrounds were compared between patients with and without MACE. To identify significant predictors of MACE, univariable and multivariable Cox proportional hazards analyses were performed. Variables for multivariable analysis were selected using a stepwise method, adhering to a minimum of 5 (ideally 10) events per variable to avoid overfitting, as previously described [23,24]. Receiver operating characteristic (ROC) analysis was employed to determine optimal cut-off values for predicting MACE and evaluate their prognostic utility. Survival analysis was also performed to evaluate the prognostic predictive ability of the determined cut-off value. All statistical tests were two-sided, with a *p*-value < 0.05 considered statistically significant.

All statistical analyses were performed using the JMP software program, version 17.2.0 (SAS Institute Inc, Cary, NC, USA).

## 3. Results

One hundred and one HCM patients were enrolled in the study. The mean age of included patients was 66 ± 11 years old, and 66 (65%) were men. All patients were followed for 64 ± 55 months after cardiac CT, and 15 patients (15%) had MACE during the follow-up period. MACE included ten hospitalizations due to heart failure, two ventricular fibrillation, two sustained ventricular tachycardia, and one sudden cardiac death. No significant differences were observed in baseline backgrounds between the groups with and without MACE (Table 1).

According to imaging parameters, patients with MACE had a significantly higher LV-ECV, left atrial diameter (LAD), LV end-systolic diameter (LVDs), and lower LVEF, than those without MACE (42 ± 8% vs. 34 ± 6%, *p* = 0.002; 48 ± 9 mm vs. 42 ± 7 mm, *p* = 0.04; 35 ± 10 mm vs. 28 ± 5 mm, *p* = 0.025; and 56 ± 13% vs. 67 ± 7%, *p* = 0.007) (Table 2).

The percentage of dilated phase HCM was significantly higher in the patients with MACE than those without MACE (40% vs. 2%, *p* < 0.001). The results of univariable Cox proportional hazards model to predict MACE are shown in Table 3.

According to univariate analysis, LVEF, LV-ECV, LAD, LVDs, and DHCM are significant predictors in MACE. Multivariable Cox proportional hazards model was performed using two variables because the number of events is small. LVEF and ECV as variables were selected by the stepwise method, which were considered important based on previous studies [25]. Multivariable Cox proportional hazards model shows LV-ECV and LVEF are both significant predictors of during the follow-up period. The hazard ratios for predicting MACE were 1.12 (95% confidence interval [CI]: 1.04–1.20, *p* = 0.003) for LV-ECV and 0.93 (95% CI: 0.88–0.98, *p* = 0.006) for LVEF (Table 4).

**Figure 3:**
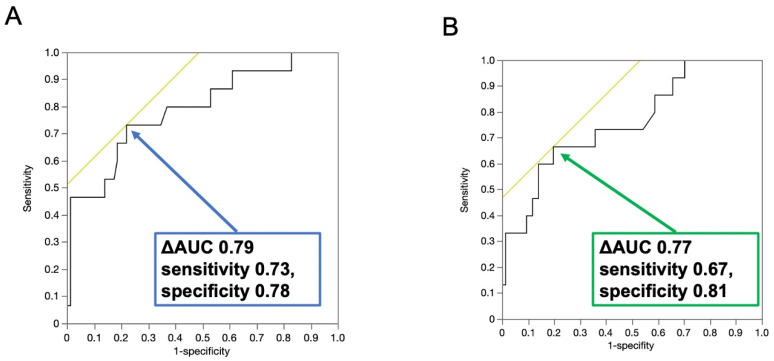
Receiver operating characteristic analysis for prediction of major adverse cardiac events. Based on the receiver operating characteristic analysis, the best cut-off value of extracellular volume fraction for the prediction of major adverse cardiac events (MACE) was 37.6%, and the sensitivity and specificity of the prediction of MACE were 73% and 78% at the cutoff of 37.6% (**A**). The best cut-off value of left ventricular ejection fraction for the prediction of MACE was 61.8%, and the sensitivity and specificity were 67% and 81% (**B**).

Based on receiver operating characteristic (ROC) analysis, the optimal threshold for LV-ECV to predict MACE was determined to be 37.6%. At this cut-off, the sensitivity and specificity were 73% and 78%, respectively, with a Youden index of 0.51. The area under the curve (AUC) for LV-ECV was 0.79 (Figure 3A). For LVEF, the optimal threshold identified by ROC analysis was 61.8%. This threshold yielded a sensitivity of 67% and a specificity of 81%, resulting in a Youden index of 0.48. The AUC for LVEF was 0.77 (Figure 3B).

**Figure 4:**
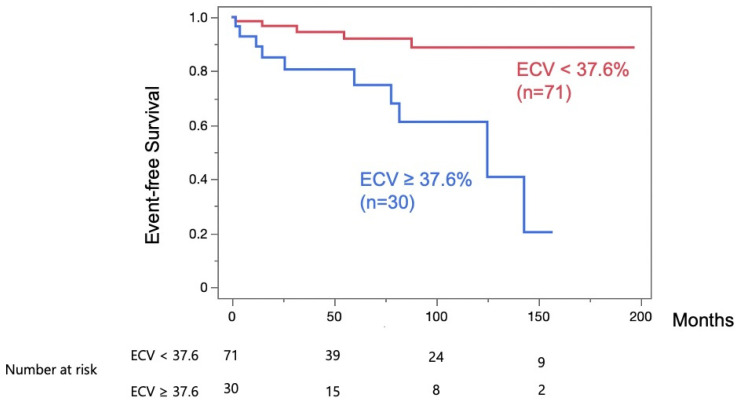
Kaplan–Meier curve of event-free survival. A significantly higher rate of major adverse cardiac events was observed in patients with left ventricular extracellular volume fraction (LV-ECV) ≥ 37.6% (*n* = 30) compared to patients with LV-ECV < 37.6% throughout the follow-up periods (*p* < 0.001).

During the follow-up periods, patients with LV-ECV ≥ 37.6% (*n* = 30) experienced significantly more MACE than those with LV-ECV < 37.6% (*p* < 0.001) (Figure 4).

The radiation dose for the additional late-phase scan was 18.6 ± 7.5 mGy as measured by the Computed Tomography Dose Index (CTDI). (Cases prior to 2015 were excluded because effective dose data had not been recorded.)

CNR of 40 patients with LE of LVM on CT was 4.0 ± 2.0.

## 4. Discussion

In this study, we evaluated the prognostic value of ECV on CT in patients with HCM. This study’s findings indicate that ECV on CT could potentially predict MACE in patients with HCM. ECV on CT was shown to be a prognostic indicator independent of cardiac function. Performing an additional late-phase scan makes ECV analysis feasible. In addition to its established roles such as assessing coronary artery stenosis and cardiac structure, CT can also be useful for quantitative evaluation of myocardial tissue characteristics. These findings indicate that CT is useful for multifaceted evaluation of cardiac assessment in patients with HCM.

For the evaluation of myocardial tissue characteristics, a visual late-phase enhancement assessment is also possible. While contrast-enhanced MRI is considered the standard method, the accuracy of late-phase enhancement evaluation in LV using CT has improved in recent years. Previously, we reported LIE on CT as a marker of MACE in cases with HCM [26]. However, in the present study, no statistically significant difference was found in the presence of LIE of LVM on CT when comparing patients with MACE to those without MACE. One of the reasons for the phenomenon is the limitation of contrast resolution of CT for the detection of LIE of LVM. Visual assessment of LIE of LVM on CT is not stable because of the low contrast in several cases, especially the border zone is difficult to detect visually. Still, ECV analysis is supportive and superior to the visual assessment of LIE, and it significantly impacts the risk assessment in patients with HCM.

### 4.1. ECV Analysis in HCM

Cardiac MRI-derived ECV of LVM has been reported to be a useful marker associated with clinical outcomes [27]. Increased ECV on LVM shows a close correlation with a higher biopsy-proven myocardial fibrosis [28]. Therefore, higher ECV means severe degeneration of the LVM, which leads to LV dysfunction or ventricular arrhythmia [29]. The prognostic value of MRI-derived ECV in HCM has been increasingly recognized. Avanesov et al. reported that the combined use of the SCD risk score and MRI-derived ECV improved the diagnostic accuracy to identify HCM patients with syncope or non-sustained VT [10]. Li et al., in a study of 243 HCM patients, demonstrated that ECV was an independent predictor of major adverse cardiac events (a composite of cardiac death, heart transplant, and aborted sudden death) [25]. In line with these findings for MRI, our study demonstrates the prognostic utility of CT-derived ECV. In contrast, Mirelis et al. previously reported no correlation between ECV on CT and ventricular arrhythmia in HCM cases with an implantable cardioverter defibrillator (ICD) [30]. However, this discrepancy may be explained by differences in the study populations; their cohort consisted exclusively of high-risk patients with ICDs, whereas our study population had a lower risk profile.

### 4.2. Comparison of CT and MRI in ECV Analysis

While MRI is the established reference standard for quantifying ECV, multiple studies have demonstrated a strong correlation between CT-derived and MRI-derived values. Two meta-analyses reported similarly high correlation coefficients: 0.90 (95% CI: 0.86–0.95) [31] and 0.89 (95% CI: 0.86–0.91) [32]. Regarding reproducibility, both modalities have shown excellent inter-observer agreement in previous reports [33,34]. We have also previously reported a strong correlation between CT- and MRI-derived ECV using the same acquisition protocol as in the present study [34]. However, each modality has a distinct profile of advantages and limitations.

MRI is contraindicated for patients with non-conditional mechanical devices or claustrophobia, and gadolinium contrast is avoided for patients with severe renal dysfunction [4]. Furthermore, acquisition time is longer, and image quality is poorer in cases with irregular heartbeats or breathing. Additionally, analysis using T1 mapping is not available at all facilities equipped with cardiac MRI because an upgrade of the latest machine or software is necessary.

Conversely, cardiac CT offers several clinical advantages. Its rapid acquisition time makes it a suitable modality for patients with arrhythmia or difficulty with breath-holding. Another key strength of CT is the ability to perform highly accurate coronary artery assessment. For HCM patients presenting with chest pain, a common clinical scenario, a single CT examination can be utilized to both evaluate for coronary artery disease and obtain prognostic data from ECV analysis. This approach of integrating myocardial tissue characterization into a standard cardiac CT protocol enhances the diagnostic yield from a single examination. Furthermore, CT serves as an essential alternative for patients with contraindications to MRI. On the other hand, CT has two main limitations. First, CT involves radiation exposure, although modern protocols minimize dose. Second, CT’s contrast-to-noise ratio is lower than MRI, which can make subtle or patchy fibrosis harder to delineate. Therefore, the choice of modality should be tailored to the individual patient’s clinical context, risk profile, and local resource availability.

### 4.3. Differences from Established Risk Factors and Clinical Utility of ECV on CT

Previous studies have reported factors such as a history of fatal arrhythmia, family history, NSVT, history of syncope, maximum wall thickness, and extensive LGE as risk factors for sudden cardiac death [35]. On the other hand, heart failure risk is thought to involve complex interactions with environmental factors, such as obesity and hypertension, which may not coincide with SCD risk factors [36]. In this study, several factors may explain why established risk factors were not significant predictors of MACE, but ECV on CT was significant.

First, our study cohort tended to consist of relatively low-risk cases with preserved LVEF and low SCD risk scores. Second, our sample size is small, and it may result in limited statistical power. Third, a large proportion of the outcomes were accounted for by heart failure hospitalizations.

Visual late-phase enhancement assessment using CT is limited in cases with subtle or diffuse myocardial fibrosis due to its low contrast resolution compared to MRI [37]. However, ECV may allow for quantitative evaluation of even subtle or diffuse fibrosis [38]. So, ECV on CT may have had significant detection ability even in this cohort with many low-risk HCM cases. Furthermore, LV-ECV reflects diffuse fibrosis, which may indicate myocardial damage caused by secondary myocardial stress. These findings suggest LV-ECV could be useful for comprehensive myocardial risk assessment from the early stages of HCM.

Most studies of ECV on CT have been observational. Prospective validation is needed before ECV on CT can guide major therapeutic decisions, such as ICD implantation. However, our findings highlight its immediate utility in risk stratification and patient management, particularly for individuals who might otherwise be overlooked. Asymptomatic or low-risk HCM patients, who are often younger, are particularly prone to discontinuing follow-up, creating a significant gap in care. The LV-ECV threshold of ≥37.6% identified in our study can address this challenge by serving as an objective, quantitative marker of risk. For clinicians, this threshold can trigger more intensive management for this subgroup, such as more frequent monitoring or proactive medication adjustments. For patients, a specific value illustrating their underlying myocardial fibrosis burden can be a useful communication tool. It helps them understand their long-term risk, thereby motivating adherence to follow-up and potentially preventing adverse events that might otherwise occur.

### 4.4. Decreased Radiation Dose and Increased Image Quality for LIE Analysis on CT

An additional radiation dose is required for ECV analysis performed using CT images. However, this is a reasonable trade-off for obtaining the useful clinical parameter. The radiation dose for the additional late-phase scan was 18.6 ± 7.5 mGy as measured by CTDI, which is comparable to standard chest CT examinations [39]. Advancements in multi-detector CT technology and new reconstruction techniques, such as iterative or deep learning reconstruction methods, have recently reduced radiation dose in late-phase cardiac imaging, while significantly improving image quality [8].

CT attenuation value increases when CT scanning is conducted with a lower tube voltage, but image noise rises because of the limited radiation dose; therefore, applying the technique to the cardiac LIE analysis on LVM has been almost impossible [40]. Meanwhile, in recent years, newer reconstruction techniques such as iterative reconstruction or deep learning reconstruction have emerged, and the maximum tube current has also improved. These technical improvements have reduced image noise in CT images taken with a lower tube voltage. Our previous report demonstrated that combining new-generation CT with iterative reconstruction enhances image quality for LIE on CT and improves its diagnostic accuracy [8]. In this study, CNR in 40 patients with LIE on CT was almost 4.0 ± 2.0. In the previous studies, the CNR of LIE on CT in cases with old myocardial infarction was close to six and four, respectively. The CNR value of LIE in the present study appears comparable to those reported in these previous studies, despite differences in the underlying myocardial disease [17,41].

### 4.5. ECV Analysis in Cases with LVH

Notably, recent improvements in the image quality, enhancing the assessment of LIE with LVM on CT, have led to the approval of CT-derived ECV analysis as an alternative to MRI in the most recent guidelines for cardiac CT and cardiac amyloidosis issued by the Japanese Cardiovascular Society [42]. Differentiating cardiac amyloidosis from HCM is often difficult in patients with LVH, because their hypertrophic morphologies on echocardiography or ST abnormalities on ECG are sometimes similar, and additional imaging techniques are necessary as usual. Therefore, cardiac CT is valuable not only for evaluating coronary artery disease because of abnormal chest symptoms or ECG, but also for the screening of cardiac amyloidosis in patients with LVH. The additional late-phase cardiac scan is helpful for their differential diagnosis because their LIE patterns are different [3]. Another study has shown that ECV analysis using CT data is useful for predicting patient prognosis in cases with cardiac amyloidosis [43]. Extending this evidence, our study in HCM patients, from whom cardiac amyloidosis had been excluded, demonstrated that ECV on CT is an independent prognostic marker. Therefore, a late-phase CT scan can provide valuable information throughout the management of patients with LVH, including both diagnosis and risk stratification.

### 4.6. Screening for Intracardiac Thrombus in Cases with AF on Late-Phase CT Images

Almost one-third of all cases in this study had AF, and late-phase CT images are helpful for evaluating myocardial fibrosis and screening luminal thrombus [19]. However, ECV analysis performed with single-energy scans might be influenced by AF or other arrhythmia, including ventricular premature beats, because of the gaps in the positions of LVM between non-contrast and late-phase images. To decrease radiation dose, non-contrast or late-phase scans were acquired using a prospective ECG gating technique that targets only limited cardiac phases (e.g., mid-diastolic). Nevertheless, in cases of AF, limiting the total scan time is challenging because predicting the timing of the next heartbeat is not possible. Therefore, scan times tend to be longer in patients with AF compared to those with normal sinus rhythm (e.g., end-systolic to mid-diastolic). To address potential motion, we selected the pair of non-contrast and late-phase images showing the fewest motion gaps from a relatively wide range of acquired cardiac phases for ECV analysis. Using this approach, we were able to report the utility of ECV analysis for predicting MACE in patients with dilated cardiomyopathy (DCM), despite approximately 20% of those cases having AF [15]. Other research groups have also reported on the utility of ECV analysis for identifying cardiac amyloidosis in patients with severe aortic valve stenosis, a population in which nearly half of the individuals had AF [44].

### 4.7. Future Perspectives

We believe that in the future, ECV on CT holds the potential for integration into risk stratification models and clinical guidelines for HCM. One approach is to combine ECV with other established markers to improve predictive accuracy. For instance, in an exploratory analysis of our data, we observed that combining LV-ECV with LVEF improved the predictive accuracy for MACE compared to LVEF alone (AUC increased from 0.76 to 0.84, sensitivity from 0.67 to 0.73, and specificity from 0.80 to 0.83). This supplementary analysis suggests that incorporating CT-derived LV-ECV as a marker of myocardial tissue characterization into established risk models such as the HCM Risk-SCD score may improve their predictive performance.

Furthermore, as the evidence base grows through larger, prospective studies, ECV on CT could be incorporated into clinical guidelines. Similarly to the recent inclusion of CT-derived myocardial assessment in the guidelines for cardiac amyloidosis, ECV on CT could become a recommended tool for risk stratification in HCM, especially for patients undergoing CT for other indications or those in whom MRI is contraindicated.

### 4.8. Limitations

Our study has several limitations that should be mentioned. First, this study was conducted as a retrospective analysis at a few centers and involved a relatively small sample size and events. Therefore, our multivariable analysis was limited to a few variables to mitigate the risk of overfitting. The results should therefore be interpreted with caution and require validation in a larger, independent cohort. Second, ECV analysis was performed using single-energy images, which needed the subtraction of late-phase and non-contrast images. Misalignment between these two phases could potentially lead to an under- or over-estimation of ECV on single-energy images when compared to analysis using dual-energy images from the latest CT scanners, which does not cause such gaps. Third, only a few high-risk HCM cases, e.g., dilated phase HCM or HOCM, were included, and forty percent of all cases were apical-type HCM in this study. The percentage of apical type HCM is relatively higher in Asian populations than in others, and apical HCM is originally thought to be lower mortality risk than others [45]. However, in recent research, it has also been reported that the mortality rate of apical HCM does not differ significantly from other types. Suggesting that the differences in subtypes may not have a significant impact on the outcome [45]. Fourthly, genetic tests have not been performed in almost all cases, and other differential diseases have not been completely ruled out. This is because, during the main follow-up period of this study, the availability of genetic testing for HCM was limited to specialized centers, which made the implementation of genetic testing in our cohort challenging. Fifth, only 33 patients in our cohort underwent both CT and MRI within one year, and in many of these cases, retrospective calculation of MRI-derived ECV was not feasible. Therefore, a direct comparison between CT- and MRI-derived ECV in this cohort was not possible. Nevertheless, previous reports have demonstrated a high correlation between CT- and MRI-derived ECV, supporting the reliability of ECV on CT as a surrogate in our study [31,32,33,34]. Finally, all patients underwent cardiac CT because of the evaluation of coronary artery diseases based on the latest guideline6, so the other patients with HCM were excluded from this analysis; therefore, the results of this study should not be applied to all HCM patients.

## 5. Conclusions

LV-ECV on CT was a valuable prognostic indicator in HCM cases and helped predict MACE in HCM cases. LV-ECV on CT could serve as a quantitative risk stratification tool, particularly for patients in whom cardiac MRI is contraindicated or not feasible.

## Figures and Tables

**Table 1:** Characteristics of patients with and without major adverse cardiac events.

	MACE (+) (*n* = 15)	MACE (−) (*n* = 86)	*p*-Value
Age, years	63 ± 16	66 ± 11	0.42
Male, *n* (%)	8 (53)	58 (67)	0.38
Body mass index, kg/m^2^	24 ± 4	25 ± 4	0.67
Atrial fibrillation, *n* (%)	7 (47)	20 (23)	0.11
Hypertension, *n* (%)	6 (40)	57 (66)	0.08
Dyslipidemia, *n* (%)	5 (33)	31 (36)	1.0
Diabetes, *n* (%)	2 (13)	17 (20)	0.73
Family history of SCD, *n* (%)	1 (7)	2 (2)	0.40
Syncope, *n* (%)	0 (0)	8 (9)	0.60
Previous NSVT, *n* (%)	5 (33)	23 (27)	0.76
eGFR, mL/min/1.73 m^2^	63 ± 21	69 ± 16	0.32
Administration of β-blocker, *n* (%)	7 (50)	36 (43)	0.77
Administration of statin, *n* (%)	5 (36)	22 (26)	0.52
Administration of ACE inhibitor or ARB, *n* (%)	4 (29)	40 (48)	0.25
Follow-up period (month)	49 ± 46	67 ± 56	0.19

SCD, sudden cardiac death; NSVT, non-sustained ventricular tachycardia; eGFR, estimated glomerular filtration rate.

**Table 2:** Parameters on transthoracic echocardiography and CT in patients with and without MACE.

	MACE (+) (*n* = 15)	MACE (−) (*n* = 86)	*p*-Value
LVEF on TTE, %	56 ± 13	67 ± 7	0.007 **
Maximum LV wall thickness on TTE, mm	19 ± 5	17 ± 4	0.18
LA diameter on TTE, mm	48 ± 9	42 ± 7	0.04 *
LVOT gradient on TTE, mmHg	7 ± 4	9 ± 14	0.31
LVOT gradient > 30 mmHg on TTE, *n* (%)	1 (7)	5 (6)	1.0
LVDd on TTE, mm	50 ± 10	46 ± 6	0.15
LVDs on TTE, mm	35 ± 10	28 ± 5	0.025 *
Valvular heart disease (≥2+)	1 (7)	2 (2)	0.38
HCM SCD risk score, %	2.4 ± 1.1	2.0 ± 1.5	0.26
Significant coronary artery stenosis, *n* (%)	3 (20)	11 (13)	0.44
LIE on CT, *n* (%)	10 (67)	40 (47)	0.17
LV-ECV on CT (%)	42 ± 8	34 ± 6	0.002 **
DHCM, *n* (%)	6 (40)	2 (2)	<0.001 **

LVEF, left ventricular ejection fraction; TTE, transthoracic echocardiography; LA, left atrium; LVOT, left ventricular outflow tract; LVM, left ventricular myocardium; ECV, extracellular volume fraction; CT, computed tomography; LVDd, left ventricular end-diastolic diameter; LVDs, left ventricular end-systolic diameter, HCM, hypertrophic cardiomyopathy; SCD, sudden cardiac death; LIE, late iodine enhancement: DHCM, dilated phase hypertrophic cardiomyopathy. * *p* < 0.05, ** *p* < 0.01. *p*-value < 0.05 was considered statistically significant.

**Table 3:** Univariate Cox proportional hazards model for predicting MACE.

Variable	Univariable		
	Hazard Ratio	95% Confidence Interval	*p*-Value
LVEF (%)	0.90	0.86–0.94	<0.001 **
LV-ECV on CT (%)	1.15	1.08–1.23	<0.001 **
LAD	1.07	1.01–1.12	0.029 *
LVDs	1.13	1.06–1.20	<0.001 **
DHCM	8.12	2.9–23	<0.001 **

HCM, hypertrophic cardiomyopathy; SCD, sudden cardiac death; LV-ECV, left ventricular extracellular volume; LAD, left atrial diameter; LVDd, left ventricular end-diastolic dimension; DHCM, dilated phase of hypertrophic cardiomyopathy. * *p* < 0.05, ** *p* < 0.01. *p*-value < 0.05 was considered statistically significant.

**Table 4:** Multivariable Cox proportional hazards model for predicting MACE.

Variable	Multivariable		
	Hazard Ratio	95% Confidence Interval	*p*-Value
LVEF (%)	0.93	0.88–0.98	0.006 **
LV-ECV on CT (%)	1.12	1.04–1.20	0.003 **

HCM, hypertrophic cardiomyopathy; SCD, sudden cardiac death; LV-ECV, left ventricular extracellular volume; LAD, left atrial diameter; LVDd, left ventricular end-diastolic dimension; DHCM, dilated phase of hypertrophic cardiomyopathy. ** *p* < 0.01. *p*-value < 0.05 was considered statistically significant.

## Data Availability

The data underlying this article will be shared on reasonable request to the corresponding author if the ethical committee agrees with the request.

## Abbreviations

The following abbreviations are used in this manuscript:

ECV	Extracellular volume fraction
CT	Computed tomography
HCM	Hypertrophic cardiomyopathy
LV	Left ventricular
LV-ECV	Left ventricular extracellular volume fraction
MACE	Major adverse cardiac events
LAD	Left atrial diameter
LVDd	LV end-diastolic diameter
LVDs	LV end-systolic diameter
LVEF	Left ventricular ejection fraction
TTE	Transthoracic echocardiography
MRI	Magnetic resonance imaging
LGE	Late gadolinium enhancement
LIE	Late iodine enhancement
LVM	Left ventricular myocardium
ECG	Electrocardiogram
HU	Hounsfield units
Hct	Hematocrit
ROI	Regions of interest
CNR	Contrast-to-noise ratio
SD	Standard deviation
LVOT	Left ventricular outflow tract
ROC	Receiver operating characteristic
CI	Confidence interval
AUC	Area under the curve
CTDI	Computed Tomography Dose Index
AF	Atrial fibrillation
DCM	Dilated cardiomyopathy
LVH	Left ventricular hypertrophy
SCD	Sudden cardiac death
NSVT	Non-sustained ventricular tachycardia
eGFR	Estimated glomerular filtration rate
DHCM	Dilated phase hypertrophic cardiomyopathy
